# Prediction of tumor grade and stage in endometrial carcinoma by preoperative assessment of sonographic endometrial thickness: Is it possible?

**DOI:** 10.4274/tjod.35651

**Published:** 2014-12-15

**Authors:** Yiğit Çakıroğlu, Emek Doğer, Şule Yıldırım Kopuk, Canan Özcan, Betül Nalbant, Aydın Çorakçı, İzzet Yücesoy

**Affiliations:** 1 Kocaeli University Faculty of Medicine, Department of Obstetrics and Gynecology, Kocaeli, Turkey

**Keywords:** Endometrial cancer, ultrasound, endometrial thickness, grade, stage

## Abstract

**Objective:**

To evaluate preopertive accuracy of endometrial thickness for assesment of histologic grade and stage of endometrial carcinoma and also determining a cut-off value for the determination of grade of endometrial carcinoma.

**Materials and Methods:**

Clinical data of 105 patients who underwent surgical staging with endometrial carcinoma were reviewed retrospectively. Preoperatively endometrial thickness were recorded and correlated with pathologic information.

**Results:**

A statistically significant correlation was found in between endometrial thickness and grade of the disease (r=0.746, p=0.001). Besides, no correlation was found between endometrial thickness and stage (r=0.153, p=0.119). The endometrial thickness at 9 mm revealed the optimal sensitivity and specificity (93.33 and 26.2, respectively) for turning through grade1 to grade 2 with 68.2% positive predictive value and 66.7% negative predictive value. We indicated the endometrial thickness at 27 mm as the optimal value with sensitivity and specificity (27.27 and 95.65, respectively) for turning through grade 2 to grade 3 with 66.7% positive predictive value and 77.5% negative predictive value.

**Conclusion:**

In conlusion, sonographic evaluation of the endometrial thickness is economical, simple and can be used as a prognostic tool for endometrial cancer grading. The operating team may have the chance to get prepared before the operation and may have the chance to inform the patient about the operation.

## INTRODUCTION

Endometrial cancer is the sixth most common cancer in women and is the most common malignancy of femal genital tract. It ranks 13^th^ in cause of death from malignancy^(1^). Incidence is rising correspondingly with increasement in obesity and life expectancy^(2,3)^. Age, histological tumor grade, depth of myometrial invasion, cervical invasion, and lymph node involvement are prognostic factors of the cancer^(4,5)^. The depth of myometrial invasion and grade are directly correlated with the prevalance of pelvic and paraaortic lymph node metastases and the 5-year survival rate^(6)^.

Histologic diagnosis of endometrial cancer is usually based on dilatation and curettage or hysteroscopic endometrial sampling. Since the pre-operative histologic diagnosis does not reflect the extent of the disease, some non-invasive diagnostic tools have been investigated for the prediction of invasion of the tumor in the literature^(7)^. Research on ultrasound (USG) and Magnetic Resonance Imaging (MRI) have been performed on patients with the diagnosis of endometrial cancer. Endometrial thickness, uterine volume, endometrial homogeneity, myometrial blood flow and intensity of the junctional zone have been investigated on USG and MRI^(8,9)^.

Endometrial cancer staging is surgical-pathological based on the International Federation of Gynecology and Obstetrics (FIGO) classification since 1988, and independent of radiological staging and assessment^(10)^. The standard surgical procedure for the treatment of endometrial cancer according to FIGO includes peritoneal cytology, total abdominal hysterectomy, bilateral salpingo-oopherectomy, and pelvic-paraaortic lymphadenectomy. In case of predicting lymph node metastases on the basis of cancer grade and myometrial invasion preoperatively, proper surgery planning of lymph node sampling and lymphadenectomy can be provided.

In this study we aimed to evaluate preopertive accuracy of endometrial thickness for assesment of histologic grade and stage of endometrial carcinoma and also determining a cut-off value for the determination of grade of endometrial carcinoma.

## MATERIALS AND METHODS

The data of 105 consecutive patients operated between September 2011 and September 2012 with the diagnosis of endometrial cancer in the postmenopausal period and who had completed surgical staging histopathologically were reviewed retrospectively. The study protocol was approved by the Ethical Committee and Institutional Review Board of Kocaeli University.

All the patients were performed ultrasound examination before endometrial biopsy. Transvaginal ultrasound (Medison Sonoace 8X Ultrasound Machine, (manufacturer), 4-8 MHz) was performed as a part of preoperative work-up a day before surgery by the same operator. Endometrial thickness was measured in the sagittal plane of the uterus at the thickest part near fundus between the endometrial-myometrial junctions on the anterior and posterior uterine walls, and any fluid in the uterine cavity was excluded. We excluded the tumor size to the endometrium that reached serosa. The mean results of three measurements were recorded.

After histopathologic confirmation of endometrial carcinoma on endometrial biopsy, all patients underwent total abdominal hysterectomy with bilateral salpingooopherectomy. Systematic pelvic and paraaortic lymph node dissection was added in the high risk patients (grade 3, unfavourable histologic type, infiltration to more than half the depth of myometrium, cervical infiltration).

We compared with ultrasound results and pathological findings by using Statistical Package for Social Sciences (SPPS) 16 software (SPSS Inc, IL., Chicago, USA) and MedCalc software version 12.3.0 (MedCalc Software, Broekstraat 52, 9030 Mariakerke, Belgium). Data are expressed as mean, standard devation and percentage. Correlation analysis was performed using Pearson correlation analysis for parametric parameters. Receiver operating characteristics (ROC) analysis was performed to detrermine a cut-off value of endometrial thickness for detection of grade. Statistical significance was considered p values than 0.05.

## RESULTS

Sociodemographic characteristics of the patients are analysed and demonstrated on Table 1. Age, body mass index, menopausal status, gravida, parity, and associated medical disorders like diabetes mellitus and hyperlipidemia are shown on the Table 1.

The intervals from ultrasound examination to endometrial biopsy and from biopsy to surgery have been evaluated. The median interval from ultrasound examination to endometrial biopsy was 5 days and from endometrial biopsy to surgery was 22 days.

Myometrial invasion, histologic subtypes, grade, and stage of the patients have been shown on Table 2. Myometrial invasion was <1/2 in 73 (69.5%) of the patients. According to our results, endometrioid adenocarcinoma has been the most common (85.7%) among the subtypes. When patients were compared according to grade, Grade 2 was the most common followed by grade 1 and grade 3 (35.2%, 43.8%, and 21%, respectively). Also, our results revealed stage 1A as the most common stage (61.9%).

On preoperative ultrasound examination, the mean endometrial thickness was 17.27±7.15 mm (4.8-35). Correlation analysis in between endometrial thickness and grade, and stage is performed. A statistically significant correlation was found in between endometrial thickness and grade of the disease (r=0.746, p=0.0001). Besides, no correlation was found between endometrial thickness and stage (r=0.153, p=0.119).

**Figure 1:**
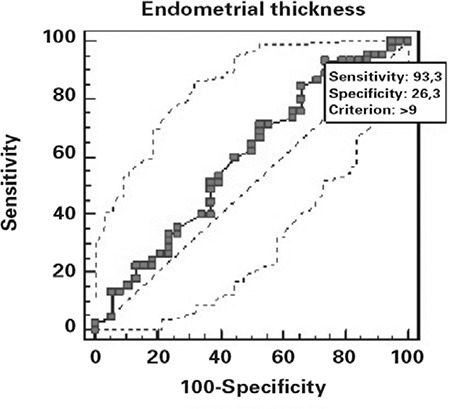
ROC curve of the endometrial thickness and grade 1 transition to grade 2 with AUC value of 0.596 (95% CI=0.482 to 0.702)

**Figure 2:**
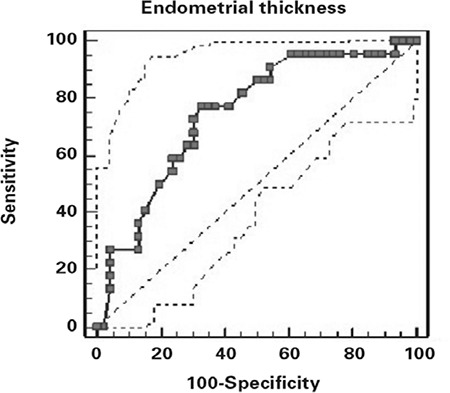
ROC curve of the endometrial thickness and grade 2 transition to grade 3 with AUC value of 0.746 (95% CI=0.625 to 0.843)

ROC curve of the endometrial thickness and grade 1 transition to grade 2 is demonstrated on Figure 1. The endometrial thickness at 9 mm revealed the optimal sensitivity and specificity (93.33 and 26.2, respectively) for turning through grade1 to grade 2 with 68.2% positive predictive value and 66.7% negative predictive value. The ROC curve of the endometrial thickness and grade 2 transition to grade 3 is demonstrated on Figure 2. We indicated the endometrial thickness at 27 mm as the optimal value with sensitivity and specificity (27.27 and 95.65, respectively) for turning through grade 2 to grade 3 with 66.7% positive predictive value and 77.5% negative predictive value.

## DISCUSSION

Grading provides a measure of tumor aggressiveness, and essential part of the FIGO staging for endometrial cancer^(10)^. Tumor grade is one of the most important prognostic factors for predicting overall survival and also most common factor that can be assessed preoperatively^(2)^.

Surgeon has to rely on the tumor grade from the endometrial biopsy which is often inaccurate and may vary the final tumor grade from the hysterectomy specimen^(8,10)^. Sanjung et al. has stated histologic grade by preoperative biyopsy with an accuracy of 89%, a sensitivity of 75% and a specificity of 95%^(9)^. However, Wang et al. have revealed an accuracy of 35.2% for histologic grade by D&C^(11)^.

Transvaginal sonography is the major non invasive diagnostic method that provides the detection of endometrial abnormalities as simple increased endometrial thickness^(12)^. The use of modern imaging tools in the preoperative assessment of staging has increasingly being recognised^(8)^. Meanwhile TV sonography is not the only diagnostic tool but also can be used as a prognostic tool. In this context, some studies have suggested ultrasound as a reliable method for detecting myometrial and cervical invasion^(13,14)^. In a study performed among 120 subjects, a sensitivity of 66% and a specificity of 72% has been stated for the detection of myometrial invasion by ultrasound^(15)^. Miklos P et al., evaluated 150 patients preoperatively and then correlated definitive histopathologic results, diagnostic accuracy of the TV sonography was obtained 82.67%, sensitivity 92.31% and specificity 79.28%^(16)^. However, Arko and Pilka have stated a limited role of ultrasound for the assessment of myometrial invasion^(17,18)^. Dueholm et al., suggested a risk of endometrial cancer (REC) scoring system based on body mass index, Doppler score, endometrial thickness and interrupted endomyometrial junction on unenhanced TVS. When REC score of ≥4, sensitivity for detection of endometrial cancer was 91% and specificity was 94%^(19)^.

MRI is another tool that has been studied for the evaluation of endometrial cancer. DelMaschio et al. have compared TV sonography and MRI in the staging diagnosis of endometrium cancer and have reported no statistical differences between the two techniques^(20)^.

We have demonstrated a correlation between endometrial thickness and grade in our study different from Eitan et al’s study(21). According to our results, endometrial thickness greater than 9 mm is able to predict a probably higher grade than grade 1, and a thickness greater than 27 mm is able to predict a probably higher grade than grade 2. In Eaten et al.’s study, no correlation was detected between endometrial thickness and grade or stage^(21)^.

Accurate preoperative staging is needed to decrease excessive or insufficient procedures especially in elderly patients^(22)^. For example, lympadenectomy might increase morbidity that can cause lymphocysts, vascular damage, as well as gastrointestinal and urogenital complications^(23)^. Even incisions may vary according tumor stage. If the gynecologist has preoperative knowledge of staging, the patient may be referred to gynecologic oncology centers.

In conlusion, sonographic evaluation of the endometrial thickness is economical, simple and can be used as a prognostic tool for endometrial cancer grading. The operating team may take a correct decision about type of surgical treatment to perform and may have the chance to inform the patient about the operation.

## Figures and Tables

**Table 1:**
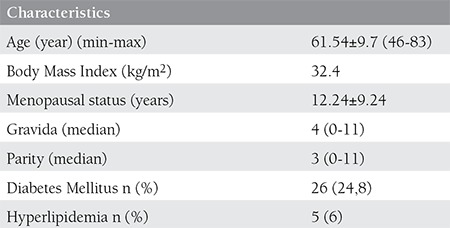
Sociodemographic characteristics of the patients

**Table 2:**
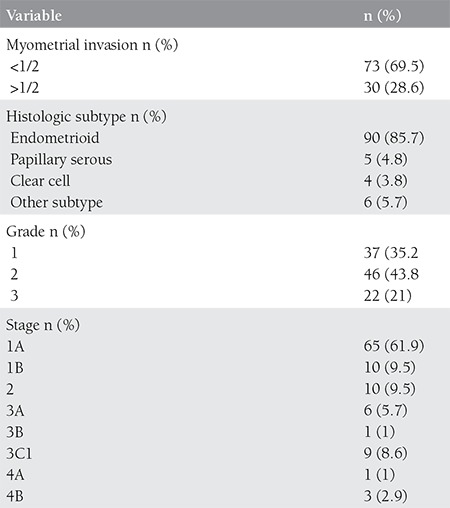
Myometrial invasion, histologic subtypes, grade, and stage of the patients
